# Supplementary material for: The health effects of hotter summers and heat waves in the population of the United Kingdom: a review of the evidence
Source: Environ Health. 2017 Dec 5;16(Suppl 1):119. doi: 10.1186/s12940-017-0322-5 (PMC5773858; doi:10.1186/s12940-017-0322-5)
Supplement: Supplementary file 1 — UK based studies examining the effects of increased temperatures on mortality. (DOCX 27 kb) [file 12940_2017_322_MOESM1_ESM.docx]

Table S1: Studies which examine the impact of heat on mortality in the United Kingdom

| Study | Study Population (time period, region, age) | Methods  and where reported threshold (either absolute °C or percentile of temperature distribution) and lag period | Exposure | Outcome(s) | Time varying confounders included in model | Results: % increase in mortality every 1 °C above threshold value (unless stated otherwise) | Comments |
| --- | --- | --- | --- | --- | --- | --- | --- |
| Hajat et al. 2002  [11] | 1976-1996  London  All ages | Time series regression  Threshold was varied between 90^th^-99^th^ percentiles of temperature distribution with little effect on the estimated RR. Results reported here are for the 97^th^ percentile (21.5 °C  ).  Lag 0 | Daily mean temperature | All-cause mortality | Season, trends over time, day of week, public holidays, influenza. | All-cause mortality 3.34%(2.47, 4.23)  Respiratory mortality 5.46% (3.43,7.52)  Cardiovascular mortality 3.01% (1.73,4.32) | Threshold choice did not appear to affect estimate of RR but threshold choice will affect number of hot days included in analysis.  Different lag periods tested (from 0-3). |
| Donaldson et al 2003  [12] | 1971-1997  South East England  All ages | Time series analysis  Comparison of change in risk over time and threshold was allowed to vary between time periods. | Daily Apparent temperature | All-cause mortality:  Daily mortality data converted into excess mortality compared to the monthly median mortality count. | Use of monthly mean as a baseline will have captured some monthly fluctuations in death rate, but no explicit control mentioned for other time-varying factors/confounders | Decrease in excess heat related mortality per 10^6 : 111(41,180) in 1971 decreased to 108 (41,176) in 1997.  After adjusting for age and sex the decrease was larger (decrease of 53 excess heat related deaths). |  |
| Pattenden et al. 2003  [10] | 1993-1996  London (study also included data from Sofia)  All ages | Time series regression  Used 90^th^ percentile of temperature distribution (21°C)to estimate heat effects and also produced estimates assuming a V shaped mortality-temperature relation (MMT) – where threshold for heat and cold days at 18°C  Lags up to 25 days assessed | Daily mean temperature | All-cause mortality | Season, trends over time, day of week, public holidays, relative humidity, particulate matter. | All-cause mortality using 90^th^ percentile as threshold 1.86% (1.36,2.36)  All-cause mortality using 18°C threshold 1.30% (0.99, 1.62)  Proportion of heat attributable deaths using 90^th^ percentile as threshold – 0.44% (0.33,0.56)  Proportion of heat attributable deaths using 18 °C as threshold – 0.82% (0.63,1.01) |  |
| Goodman et al. 2004  [13] | 1980-1996  Dublin  All ages | Time series regression  Threshold not reported  Lag 0 | Daily minimum temperature | All-cause mortality (excluding external causes) and cardiovascular and respiratory mortality | Season, trends over time, humidity, particulate pollution | All-cause mortality 0.4% (0.1,0.6)  Cardiovascular mortality 0.0% (-0.4, 0.4)  Respiratory mortality 0.8% (0.1,1.5) | Effect was also larger in older age groups |
| Hajat et al., 2005  [14] | 1991-1994  London (study also included data from Delhi and Sao Paulo)  All ages and by age category | Time series regression  Threshold 20 °C  Lag 0-1 but then examined deficits of deaths at longer lags to examine mortality displacement | Daily mean temperature | All-cause mortality and split by cause (cardiovascular, respiratory) | Season, trends over time, humidity, rainfall, particulate pollution, influenza A | Lag 0  All-cause mortality 1.4 (0.8, 2.0)  Lag0-1 week  All-cause mortality 0.9 (-0.2,2.0)    Lag 0-4 weeks  -1.6 (-3.4,0.3) | Sum of the heat effects by day 11 was 0 |
| Carson et al. 2006  [15] | 1900-1996  Time periods analysed in 10 year bands to assess the change in heat related risk over time  London  All ages | Time series regression  Threshold 15°C | Mean weekly temperature | All-cause weekly mortality and by cause: cardiovascular and respiratory mortality | Trends over time and seasonality. excluded years of war and influenza pandemic  For later period also included air pollution (PM10) | RR of all-cause mortality and % attributable deaths  1900-1910 1.02(-0.16,.21) 0.4% (-0.06,0.86)  1927-1937 1.53(0.152.93) 0.89%(0.09,1.69)  1954-1964 0.29(-1.95,2.59) 0.06% (-0.39,0.5)  1986-1996 -1.34(-1.94,-0.75)  -0.9%(-1.31,-0.5) | Risk of heat related death decreased over time. Was highest in the period 1927-1937 and lowest between 1986-1996  The weekly data used meant that results may have been attenuated for heat affects |
| Hajat et al 2007.  [16] | 1993-2003  10 governmental regions of England and Wales  Mortality data linked to census data to investigate sub-groups at risk (by, deprivation, persons living alone or nursing homes, sex etc.) | Time series regression  Lag 0-1  Region specific thresholds selected. | Daily mean temperature | All-cause mortality | Season, trends over time, day of week, public holidays, ozone, influenza. PM10 | All-cause mortality 3% (2.0,3.0) | Little effect modification by deprivation (as tested by quintile of deprivation)  Effect stronger in urban compared to rural locations  Those in nursing homes were at increased risk  Women over 65 yrs at increased risk  Deaths from respiratory and external causes most strongly associated with heat exposure |
| Page et al. 2007  [17] | 1993-2003  England and Wales  All ages | Time series regression used for overall heat effect. Also episode analysis for the effect of heat waves.  Threshold 18°C | Daily mean temperature (Central England Temperature) | Suicide | Season, trends over time, day of week, public holidays, length of daylight | Suicide 3.8 % (2.1,5.6) | The increase in violent suicide was greater than non-violent suicide.  Percentage increase of male and female suicide above the threshold was similar. |
| Baccini et al. 2008  [18] | 1990-2000 (Dublin)  1992-2000 (London)  London, Dublin (study included data from 14 European studies)  All ages and by age category | Time series regression  Warm season only  Threshold:  London 23.9 °C (22.6,25.1)  Dublin 23.9 °C (20.7,27.1)  Lag 0-3 | Apparent temperature | All-cause mortality (excluding external causes) | Holidays, day of the week, calendar month, barometric pressure, wind speed, NO2 | London 1.54 % (1.01, 2.08)  Dublin -0.02 (-5.38, 5.65) | Harvesting was assessed – the cumulative effect of heat was only 30% of that at lag 0-3 compared to at lag 25 |
| Ishigami et al 2008  [19] | 1993-2003  London (study included 3 European cities)  All ages, split also into age category | Time series regression  Threshold 95^th^ percentile  Lag 0-1 | Daily mean temperature | All-cause mortality and by cause (cardiovascular, respiratory and external) | Season, trends over time, day of week, PM10, O3 | Age <75 years:  All cause 3% (2.0 ,5.0)  CVD 3% (1.0,4.0)  Respiratory 5% (1.0,8.0)  External 6% (2.0,10.0)  Age > 75 years:  All cause 6% (5.0,7.0)  CVD 6% (5.0,7.0)  Respiratory 8% (6.0,10.0)  External 10% (2.0, 18.0) | Heat effects greater in females across age categories  No obvious effect modification by deprivation (assessed by deprivation quintile) |
| Pattenden et al. 2010  [20] | 1993-2003  15 conurbations in England and Wales  All ages | Time series regression  Summer only (May-Sept): Risk at 97.5^th^ percentile of summer temperatures compared to 75^th^ percentile  2 day mean of daily max temperature used | maximum temperature |  | Season, trends over time, day of week, PM10, O3 | All ages:  All cause: 7% (5.0,9.3)  CVD 5.5% (2.5,8.7)  Respiratory 13.9% (7.9,20.2)  All cause:  Age 65-74 6.7 (3.1,10.3)  Age 75-84 9.2% (6.3,12.1)  Age >85 12.4% (9.3,5.5) | Risk increased with age.  There was an interaction between the effect of heat and ozone in London . |
| Armstrong et al. 2011  [21] | 1993-2006  10 governmental regions of England and Wales  All ages | Time series regression.  Thresholds allowed to vary by region but also results presented for threshold at the 93^rd^ percentile  Lag 0-1 | Daily mean temperature | All-cause mortality | Season, trends over time, day of week, humidity | Overall effect 2.1% (1.6,2.6)  NE 0.8% (0.2,1.3)  NW 1.3% (1.0,1.6)  Yorks & Humberside 1.7% (1.3,2.1)  Wales 2.0%(1.5,2.5)  W Mids 2.2% (1.9,2.6)  East Mids 2.3% (1.9, 2.8)  SW 2.1% (1.7,2.5)  SE 2.6% (2.2, 2.9)  East 2.4% (2.0,2.8)  London 3.8% (3.4,4.1) | Regions with higher temperatures had higher thresholds (in absolute °C – the threshold was at the 93^rd^ percentile of the regional temperature distribution across the regions)  Regions with higher summer temperatures showed greater increases in risk above the threshold |
| Gasparrini et al. 2012  [22] | 1993-2006  10 governmental regions of England and Wales  All ages | Time series regression  Summer months  Threshold 93^rd^ percentile of temperature distribution  Lag 0-1 | Maximum daily temperature | Mortality by cause:  cardiovascular mortality, endocrine mortality, respiratory, neurological mortality and genito-urinary causes | Trends over time, day of the week, within summer seasonal variation | % increase per °C increase above threshold:  All cause 2.1 % (1.6,2.6)  Selected causes:  Cardiovascular diseases 1.8% (1.2,2.5)  Stroke 2.5% (1.6,3.4)  Ischaemic heart disease 1.7% (1.2,2.2)  Myocardial infarction 1.1% (0.7,1.5)  Pulmonary heart disease 8.3% (2.7,4.3)  Respiratory deaths 4.1% (3.5,4.8)  COPD 4.3%(3.6,5.1)  Asthma 5.5% (2.8,8.3)  Respiratory infections 4.2% (3.5,5.0)  Endocrine 2.9% (1.7,4.2)  Genito-urinary system 3.8% (2.9,4.7)  Nervous system 4.6% (3.7,5.4)  Heat deaths attributable to specific causes (n and %) :  Cardiovascular 8005 (33.9%)  Stroke 2864 (12.1%)  Ischaemic heart disease 3725 (12.1%)  Myocardial infarction 1121 (4.1%)  Pulmonary heart disease 37 (0.2%)  Respiratory deaths 5841 (24.1%)  COPD 1821 (7.7%)  Asthma 133 (0.6%)  Respiratory infections 3194 (13.5%)  Endocrine 446 (1.9%)  Genito-urinary system 732 (3.1%)  Nervous system 1118 (4.7%) | Heat slopes higher for London and other regions with higher mean temperatures. |
| Page et al 2012  [23] | 1998-2007  UK patients registered on the UK General Practice Research Database (GPRD) with psychoses, dementia, alcohol misuse and other substance misuse  All ages | Time series regression  Threshold 93^rd^ percentile of temperature distribution  Lag 0 | Average daily temperature | Mortality (all-cause) in this subgroup of patients) | Trends over time, season, humidity, day of week | Overall effect  4.9% (2, 7.8) | Effect modifiers – age and diagnosis: those under 65 were at greatest risk and those with primary diagnosis of alcohol misuse or other substance misuse  Effect size was largest in the South East and East midlands |
| Baccini et al 2013  [24] | 1990-2000 (Dublin)  1992-2000 (London)  London, Dublin (study included data from 14 European studies)  All ages | Attributable deaths from the PHEWE study (Baccini et al 2011) were translated into Years of Life Lost (YLL) | Daily maximum apparent temperature | YLL | As per Baccini et al 2008 | YLL attributable to heat before accounting for harvesting (across all ages):  London 1914 (1033,2841)  Dublin 7 (1,15)  YLL attributable to heat after accounting for harvesting (across all ages):  London 356 (219,498)  Dublin 1 (0,2) | Estimates were adjusted for the ‘harvesting’ effect.  Study found that harvesting had a greater effect on results in the North continental than Mediterranean cities. This could reflect differences in frailty between populations, or a lack of further recruitment of frail individuals into the high risk pool in the North continental region. Also, the thresholds for models were lower in North continental regions – more frail individuals could be more sensitive to lower threshold and therefore harvesting in the Mediterranean could have been under-estimated. |
| Bennet et al. 2014  [25] | 2001-2010  376 local authority districts in England and Wales  All ages and by age category | Time stratified case-crossover  District specific thresholds at 85^th^ percentile of temperature distribution (based on summer temperatures – range of thresholds used from 15.4 °C to 19.9 °C)). Results also analysed using a common threshold of 18 °C. | Daily Mean Temperature | Cardio-respiratory mortality | Control days on same day of week selected for each death and same calendar month. PM10 controlled for | National level increase in OR of cardiorespiratory mortality:  Men:  <75 yrs 2.7 (1.6,3.8)  75-84 yrs 2.2 (1.1,3.2)  >85 yrs 2.4 (1.2,3.6)  Women:  2.4 (1.0,3.9)  3.4 (2.3,4.6)  3.9 (3.0,4.8)  Effects of hot weather varied across districts#; London, South and South East were most affected | Deprivation measure on the Carstairs score – combines indicators of unemployment, crowding of housing, social class and vehicle ownership.  Variables of greenspace and of urban/rural residence also assigned to deaths. |
| Vardoulakis et al. 2014  [26] | 1993-2006  10 governmental regions of England and Wales  All ages | Time series regression  Threshold 93^rd^ percentile  Summer months only analysed  Lag 0-1 | Daily mean temperature | All-cause mortality (including external causes) | Trends over time, season, humidity, day of week | All-cause mortality 2.5% (1.9,3.1) |  |
| Guo et al. 2014  [27] | 1993-2006  10 governmental regions of England and Wales (study included data from 12 countries/regions)  All ages | Time series regression  Estimates are for the 99^th^ vs 80^th^ percentile of temperature distribution and for 99^th^ vs 90^th^ percentile  Lag 0-21 days (heat and cold effects modelled) | Daily mean temperature | All-cause mortality (excluding external causes) | Trends over time, season, , day of week | All-cause mortality 99^th^ vs 80^th^ percentile:  5% (4.0,6.0)  All-cause mortality 99^th^ vs 90^th^ percentile:  3% (3.0, 4.0) | Minimum mortality temperatures were estimated to be around the 75^th^ percentile of the country-specific temperature distribution for all countries included in the study |
| De’Donato et al. 2015  [28] | 1996-2002 and 2004-2010  London (study included data form 9 European cities) | Time series regression.  Purpose of study was to compare risk of mortality before and after the heatwave of 2003 (in 2004 the HW plan was introduced).  Estimates are for the 75^th^ vs 99^th^ percentile of temperature distribution | Daily mean temperature | All-cause mortality (excluding external causes)  And deaths attributable to heat | Trends over time, season, humidity, wind speed, barometric pressure, NO2 | 1996-2002  All cause 2 %( 1.6,2.5)  2004-2010  All cause 1.8% (1.12,1.23) | No significant difference in the estimated relative risk of mortality above the threshold temperature for the two time periods.  Some cities in the study did have a reduced risk in the later time period (Athens, Rome, Paris) |
| Gasparrini et al 2015  [29] | 1993-2006  10 governmental regions of England and Wales (study included data from 12 countries/regions)  All ages | Time series regression (DLNM).  Threshold (MMT) 90^th^ percentile of temperature distribution.  Location specific RR were pooled using multivariate meta-analysis.  Attributable deaths and fraction of attributable deaths calculated from RR and contribution of days in the series above the MMT. Contribution of extreme heat also assessed (temperatures greater than the 97.5^th^ percentile) | Daily mean temperature | Deaths attributable to heat | Trends over time, season, , day of week | All-cause mortality:  Fraction of all deaths attributable to heat 0.30% (0.25,0.36) | MMT percentile ranges between 80^th^-90^th^ percentiles of the temperature distribution for each region |
| Gasparrini et al. 2015  [30] | 1993-1999  2000-2006  10 governmental regions of England and Wales (study included data from 12 countries/regions)  All ages | Time series regression (DLNM).  Threshold (MMT) 73^rd^ percentile. RR at the 90^th^ percentile vs 73^rd^ percentile estimated  Analyses restricted to summer period | Daily temperature | All-cause mortality | Trends over time, season, , day of week | RR at 90^th^ vs 73^rd^ percentile of temperature distribution  1993-2006 1.006 (0.993,1.019)  1993 1.005 (0.983,1.027)  2006 1.014 (0.995,1.032)  RR at 99^th^ vs 73^rd^ percentile of temperature distribution  1993-2006 1.167 (1.108,1.230)  1993 1.158 (1.093,1.227)  2006 1.168 (1.111,1.229) | Difference in RR between the two time periods not significant for the UK (but a significant reduction in risk was seen for Japan, Spain and the US – p<0.001) |
