# Supplementary material for: The health effects of hotter summers and heat waves in the population of the United Kingdom: a review of the evidence
Source: Environ Health. 2017 Dec 5;16(Suppl 1):119. doi: 10.1186/s12940-017-0322-5 (PMC5773858; doi:10.1186/s12940-017-0322-5)
Supplement: Supplementary file 2 — UK based studies examining the effects of increased temperatures on morbidity. (DOCX 19 kb) [file 12940_2017_322_MOESM2_ESM.docx]

Table S2: Studies which examine the impact of heat on morbidity outcomes (health outcomes other than mortality) in the United Kingdom

| Study | Study Population (time period, region, age) | Methods  And where reported threshold (either absolute DC or percentile of temperature distribution) and lag period | Exposure | Outcome(s) | Time varying confounders included in model | Results: increase in morbidity outcome every 1 °C above threshold value (unless stated otherwise) | Comments |
| --- | --- | --- | --- | --- | --- | --- | --- |
| Kovats et al. 2004  [32] | 1994-2000  Greater London  All ages and by age category | Time series regression  Modelled above a threshold – threshold varied by cause of hospital admission (12 °C for all-cause admissions)  Lag 0-2 days | Daily mean temperature | Emergency hospital admissions – all cause and cause specific (cardiovascular, respiratory, cerebrovascular, renal – acute renal failure and kidney stones) | Long term trend, season, public holidays, day of week, relative humidity, pollution (ozone and PM10) | All cause -0.4% (-0.22, 0.13)  Cardiovascular 1.71% (-2.70,6.33)  Respiratory 5.44% (-1.55,-0.21)  Renal 1.30% (0.27,2.35)  Respiratory > 75 yrs 10.86% (4.44,17.67)  During 1995 HW, small increase in hospital admissions – 2.6% (96% CI -2.2, 7.6) | Thresholds varied by cause of hospital admission (e.g. all cause – threshold at 12 °C, respiratory threshold was 23 °C). Risk of respiratory admission was increased with hot temperatures for all age categories. |
| Atherton et al. 2005  [34] | 1998  Leicester (population admitted to Leicester Royal Infirmary)  All ages and by age group (adult, paediatric) | Time series regression  No information on threshold or lag | Maximum and minimum temperature | Total trauma admissions, adult and paediatric trauma admissions, adult neck of femur (NOF) admissions | Season, day of week, weekends and public holidays, month, school holidays | For each 5 °C rise in Max temperature Incidence Rate Ration (IRR):  All trauma admissions 1.03 (0.99,1.07)  Paediatric trauma admissions 1.11 (1.03,1.19).  For each 5 °C rise in Min temperature IRR:  Paediatric trauma admissions 1.24 (1.12,1.38). |  |
| Lee et al. 2008  [39] | 1988-2000  London  All births on the | Time series regression  Lag 0-6 | Daily maximum temperature  Exposure defined in relation to birth (i.e. temperature in 6 days preceding birth, rather than exposure in a given trimester or stage of pregnancy) | Preterm birth (occurring <37 weeks gestation) | Long term trend, season, public holidays, day of the week, PM_10_, ozone | No evidence of an association between increased temperatures in the 6 days prior to birth and pre-term birth. |  |
| Bhaskaran et al. 2010  [36] | 2003-2006  15 conurbations in England and Wales | Time series regression  Lag 0-1 | Daily mean temperature | Myocardial infarction (MI) : all events with diagnosis of ST elevation and non-ST elevation MI on discharge or positive troponin | Long term trend, season holidays, day of week, influenza, respiratory syncytial viruses, PM_10_ , ozone | No evidence of an increased risk of myocardial infarction with heat |  |
| Parsons et al. 2010  [35] | 1996-2006  England and Wales  All ages and by age group (adult and paediatric) | Time series regression  No information on threshold or lag | Daily minimum and maximum temperature | Total adult and paediatric trauma admissions | Season, day of week, public and school holidays, year, | For each 5 °C rise in Max temperature and increase in adult trauma admissions of 1.8% and of paediatric admissions of 10%. | No confidence intervals or significance reported |
| Thornes et al. 2014  [38] | 2007-2011  Birmingham  All ages | Compares daily temperature with ambulance call out data, but not as a full time series regression analysis (looks at correlation only without adjustment for other time varying factors). | Daily minimum, mean and maximum temperatures | Ambulance call outs and response times (categorised as total number of 999 calls for one day, total category A calls (life threatening) % of category A calls responded to within 8 mins, average travel time to arrive at incidences | None | Observed reduction in % of category A responses within 8 minutes at increasing temperatures (not quantified).  During the 2003 heatwave there was a linear (positive) relationship between increasing maximum temperature and 999 calls. | Daily ambulance calls can be affected by the number of ambulances and staff on duty, business of A and E departments, road conditions, call volume and distance to the event which precipitated the call out |
| Bhaskaran et al. 2014  [37] | 2003-2009  11 conurbations in England and Wales | Time-stratified case crossover  Threshold 20°C  0-6 hours | Hourly temperature | Myocardial infarction (MI) : all events with diagnosis of ST elevation and non-ST elevation MI on discharge or positive troponin | Nitrogen dioxide, relative humidity, public holidays, day of the week, seasonality | Odds ration (OR) for MI at 1-6 hours:  1.019 (1.005,1.033) | Effect at 0-6 hours but not seen at longer lags is likely due to short term displacement.  No significant effect modification seen by individual level factors such as whether patients had previous heart disease, were taking aspirin or had previous hypertension. |
